# Early occurrence of primary angle-closure glaucoma in a patient with retinitis pigmentosa and *CRB1* gene variations

**DOI:** 10.5935/0004-2749.20220078

**Published:** 2023

**Authors:** Ricardo Yuji Abe, Luciana de Sá Quirino Makarczyk, Marcos Pereira de Ávila, Juliana Maria Ferraz Sallum

**Affiliations:** 1 Hospital Oftalmológico de Brasília, Brasília, DF, Brazil.; 2 Department of Opthalmology, Universidade Federal de Goiás, Goiânia, GO, Brazil.; 3 Department of Opthalmology, Escola Paulista de Medicina, Universidade Federal de São Paulo, São Paulo, SP, Brazil.

**Keywords:** Glaucoma, angle-closure, Retinitis pigmentosa/diagnosis, Macular edema, Gonioscopy, Genetic testing, Humans, Case report, Glaucoma de ângulo fechado, Retinite pigmentosa/ diagnóstico, Edema macular, Gonioscopia, Testes genéticos, Humanos, Relatos de casos

## Abstract

We describe the case of a 15-year-old girl with decreased visual acuity associated with elevated intraocular pressure in both eyes and angle closure on gonioscopy. She also presented attenuation of retinal vessels and optic disc pallor with large excavation in the left eye. Ultrasound biomicroscopy revealed an anteriorly positioned ciliary body and absence of ciliary sulcus, confirming the plateau iris configuration. Spectral-domain optical coherence tomography revealed a bilateral cystoid macular edema. Genetic screening revealed heterozygous variants of the Crumbs homolog 1 (*CRB1*) gene (c.2843G>A and c.2506C>A). The patient underwent trabeculectomy for intraocular pressure control and topical treatment for macular edema. This case highlights the importance of performing gonioscopy and evaluating intraocular pressure in patients with a shallow anterior chamber despite young age. In addition, it also shows the importance of genetic screening, when available, in elucidating the diagnosis and providing patients and their families’ information on the patient’s prognosis and possible therapeutic options.

## Abstract (pt)

Nós descrevemos um caso de uma paciente de 15 anos com queda de acuidade visual e aumento da pressão intraocular em ambos os olhos, juntamente com fechamento angular no exame de gonioscopia. Na fundoscopia a paciente apresentava atenuação dos vasos retinianos, palidez de disco e aumento de escavação em olho esquerdo. Ao exame da biomicroscopia ultrassônica, foi evidenciado corpo ciliar anteriorizado e ausência de sulco ciliar em ambos os olhos, relevando presença de íris em plateau. Ao exame de tomografia de coerência óptica, visualizamos presença de edema macular cistoide bilateral. O *screening* genético revelou heterozigose no gene *CRB1* (c.2843G>A and c.2506C>A), confirmando o diagnóstico de retinose pigmentar. Este caso reforça a importância do exame de gonioscopia e da avaliação da pressão intraocular em pacientes em câmara rasa, mesmo em pacientes jovens. Além disso, mostra a importância do *screening* genético como ferramenta útil para elucidação diagnóstica.

## INTRODUCTION

Angle-closure glaucoma is not common in young individuals because the principal mechanism of primary angle closure is relative pupillary block that can increase with aging owing to the phacomorphic component in susceptible eyes^(1)^. Thus, in the management of young patients with primary angle-closure glaucoma (PACG), causes other than relative pupillary block, such as plateau iris syndrome, iridociliary cysts, previous history of retinopathy of prematurity, uveitis, and nanophthalmos, must be investigated.

Retinitis pigmentosa (RP) is described as a group of hereditary retinal diseases caused by cone rod and photoreceptor degeneration. The disease has been associated with different patterns of inheritance, such as the autosomal-dominant, recessive, or X-linked trait. In these patterns, mutations in the gene Crumbs homolog 1 (*CRB1*) have been previously described^(2)^. In contrast to the common manifestation in RP, *CRB1* retinitis is more heterogeneous and has a later age of onset. *CRB1* retinitis is associated with “Coats-like” exudative vasculopathy and relative preservation of the para-arteriolar retinal pigment epithelium^(3)^.

The odds of developing PACG are greater in patients with RP than in the general population. Patients with mutations in *CRB1* may also present hypermetropia and a short axial length, which could increase the risk of developing angle-closure glaucoma^(4)^. We present an unusual case of early-onset PACG caused by plateau iris syndrome and RP.

## CASE PRESENTATION

A 15-year-old Caucasian girl complained of poor vision in left eye despite the use of glasses. On examination, the logMAR best-corrected visual acuity was 0.1 in the right eye and 0.4 in the left eye. The static refractive error (spherical equivalent) was +2.25 diopters for the right eye and +0.5 diopters for the left eye. No nystagmus was observed, the anterior chamber depth was shallow, and no relative afferent pupillary defect was detected. Goldmann applanation tonometry values were 19 and 40 mmHg in the right and left eyes, respectively. Gonioscopic examination with a Posner goniolens revealed appositional and synechial angle closures in the right and left eyes, respectively, without double hump sign.

**Figures 1:**
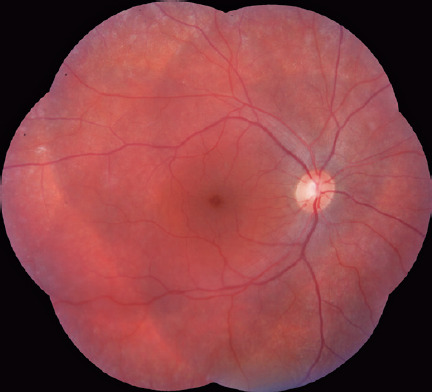
Retinography image (Visucam 524, Carl Zeiss Meditec, Inc, Dublin, CA) showing the right eye with normal optic disc color and excavation with subtle foveal and parafoveal pigmentary changes. Retinal pigment epithelial mottling can be observed in the periphery.

**Figures 2:**
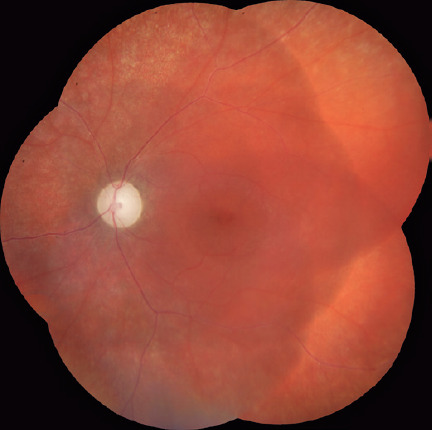
Retinography image (Visucam 524, Carl Zeiss Meditec, Inc, Dublin, CA) of the left eye showing optic disc pallor with large excavation and attenuation of the retinal vessels with no intraretinal pigmentation (bone-spicule deposits). Foveal pigmentary changes and retinal pigment epithelial mottling can be observed in the periphery.

Dilated fundus examination and retinography (Visucam 524, Carl Zeiss Meditec, Inc, Dublin, CA) revealed normal optic excavation and subtle foveal and parafoveal pigmentary changes in the right eye (Figure 1). In the left eye, we observed attenuation of the retinal vessels, with no intraretinal pigmentation (bone-spicule deposits) and optic disc pallor with large excavation (Figure 2). Visual fields were obtained using the Humphrey Field Analyzer II (model 750i; Carl Zeiss Meditec, Inc) and showed diffuse loss of retinal sensitivity, with central island of vision in the left eye. The axial length measured using optical biometry (IOL Master 700, Carl Zeiss Meditec, Inc) was 21.07 mm in the right eye and 20.48 mm in the left eye, and the anterior chamber depths were 2.73 and 2.58 mm in the right and left eyes, respectively. Ultrasound biomicroscopy (Vumax, Sonomed, New Hyde Park, NY) revealed an anteriorly positioned ciliary body, supporting the peripheral iris, along with absence of any iris cyst and ciliary sulcus.

**Figure 3:**
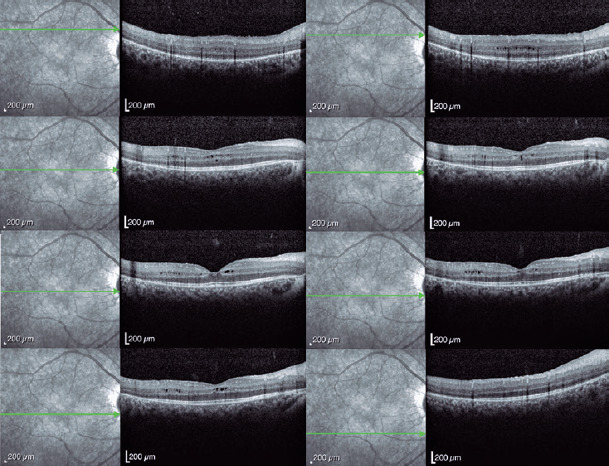
Macular spectral-domain optical coherence tomography (SD-OCT) image (Spectralis; Heidelberg Engineering GmbH, Heidelberg, Germany) showing a cystoid macular edema in the right eye. The cystoid spaces are mainly located in the inner and outer nuclear layers.

**Figure 4:**
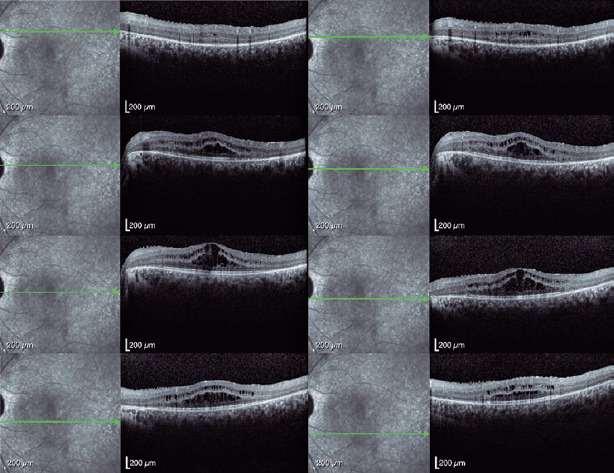
Macular spectral-domain optical coherence tomography image (Spectralis; Heidelberg Engineering GmbH, Heidelberg, Germany) of the left eye showing hyper-reflective foci in all retinal layers with higher density adjacent to the cystoid spaces at the inner and outer nuclear layers and along the outer plexiform layer. No parafoveal interdigitation and ellipsoid zones with thinning of the photoreceptor outer segments can be observed.

Spectral-domain optical coherence tomography (Spectralis, Heidelberg Engineering GmbH, Heidelberg, Germany) revealed a bilateral cystoid macular edema that was most prominent in the left eye (Figures 3 and 4). Genetic screening revealed heterozygous variants in the *CRB1* gene (c.2843G>A and c.2506C>A), confirming the diagnosis of RP. The patient´s intraocular pressure (IOP) remained high, despite maximum medical treatment and the angle remained closed despite YAG laser iridotomy. Trabeculectomy was performed in the left eye to achieve adequate IOP control.

We examined the patient’s younger brother (8 years old), who also presented a bilateral cystoid macular edema. Gonioscopic examination revealed an appositional angle closure with an IOP of 19 mmHg in both eyes under hypotensive topical medication. Genetic testing of the sibling also revealed heterozygous variants in the *CRB1* gene (c.2843G>A and c.2506C>A). We also examined her parents, and none of them presented any abnormality in the ophthalmologic examination.

## DISCUSSION

We describe the case of a patient with early occurrence of PACG induced by plateau iris syndrome associated with RP and *CRB1* gene mutation. The present case highlights the importance of prompt diagnosis and treatment of elevated IOP and angle evaluation in patients with a shallow anterior chamber, despite young age, to minimize optic nerve damage. It also demonstrates the importance of complete evaluation of macular abnormities with genetic screening to provide parents with proper guidance and information on the prognosis of the disease.

The *CRB1* gene can participate in promoting the axial growths of the head and eye during development^(5)^. In fact, high hyperopia with or without nanophthalmos is commonly found in patients with *CRB1* mutations, corroborating the findings that the *CRB1* gene not only is associated with retinal dystrophies but also may be involved in ocular abnormalities related to axial length^(3)^. Recently, Liu et al. applied whole-exome sequencing to examine patients with early occurrence of PACG with or without RP^(6)^. When comparing patients with PACG alone and patients with PACG and RP, no significant differences were reported regarding biometric parameters. Liu et al. did not indicate in detail whether plateau iris syndrome was present as a phenotype in their sample. Their results indicated that the c.1841G>T in *CRB1* was associated with the combination of RP and glaucoma, which was different from the results presented in our patients (c.2843G>A and c.2506C>A). Data are scarce regarding the association between mutations in the *CRB1* gene and PACG secondary to plateau iris syndrome.

The association between CRB1 retinopathy and glaucoma was previously described by Talib in 2017^(7)^. They investigated the clinical characteristics of 55 patients with retinopathy and *CRB1* gene mutation and found that 14% of the patients (7/55) presented with glaucoma. Among these patients, 71% had PACG. However, they did not investigate the mechanisms of the angle closure in the patients in their study. Waseem et al. identified a gene that can be associated with the development of primary angle closure in a British family by using genetic linkage^(8)^. Of the affected individuals, plateau iris configuration was observed on gonioscopy. One limitation of the present study is that we did not investigate genes related to PACG. To date, no association was found between *CRB1* gene mutation and iris and ciliary body changes associated with plateau iris syndrome.

Unfortunately, at the time of diagnosis, our patient presented significant damage to the optic nerve due to previous high IOPs. This was more critical because the macular edema from the RP decreases the quality of the central remaining vision. Nevertheless, with genetic screening, the family could obtain the exact diagnosis of the retinopathy, and this information will help to better follow the patient and her sibling, minimizing the risk of developing optic nerve damage secondary to glaucoma and monitore the macular edema properly.

For some patients with macular edema secondary to RP, combination of treatment with topical nonsteroidal anti-inflammatory and carbonic anhydrase inhibitors can represent a treatment option, even though more studies are necessary to confirm its efficacy. Until now, only patients with *RPE65*-mediated inherited retinal dystrophy can benefit from gene therapy approaches^(9)^. Gene therapy for *CRB1* retinopathy has been attempted in animal models. However, therapy for the human disease is complex, as the *CRB1* gene is a structural and signaling transmembrane protein present in three cell classes, namely Müller glia, cone, and rod photoreceptors.

In summary, our case report highlights the importance of evaluating IOP and the optic nerve and performing gonioscopy examination to rule out the possibility of PACG in patients with RP with *CRB1* gene mutation. In addition, for young patients with unexpected retinal and macular changes, our findings reinforced the use of genetic screening as an important diagnostic tool.
